# Quality indicators for safe and effective use of medications in long‐term care settings: A systematic review

**DOI:** 10.1002/bcp.70242

**Published:** 2025-08-18

**Authors:** Daria S. Gutteridge, Annabel H. Calder, Jacquelina Stasinopoulos, Sara Javanparast, Gillian E. Caughey, Jodie B. Hillen, Andrew C. Stafford, Gregory M. Peterson, Maria C. Inacio, Jyoti Khadka, Lisa M. Kalisch Ellett, Shane L. Jackson, Peter D. Hibbert, Monica L. Cations, Megan E. Corlis, Solomon C. Yu, Malcom J. Clark, Natalie R. Soulsby, Elizabeth Manias, Grace H.‐Y. Yoo, Janet K. Sluggett

**Affiliations:** ^1^ University of South Australia, UniSA Allied Health and Human Performance Adelaide SA Australia; ^2^ Registry of Senior Australians Research Centre South Australian Health and Medical Research Institute Adelaide SA Australia; ^3^ Registry of Senior Australians Research Centre, Caring Futures Institute, College of Nursing and Health Sciences Flinders University Bedford Park SA Australia; ^4^ School of Pharmacy and Pharmaceutical Sciences, Faculty of Health, Medical and Behavioural Sciences University of Queensland Brisbane QLD Australia; ^5^ Curtin Medical School, Faculty of Health Sciences Curtin University Perth WA Australia; ^6^ University of Tasmania, School of Pharmacy and Pharmacology Hobart TAS Australia; ^7^ Health and Social Care Economics, Caring Futures Institute Flinders University Adelaide Australia; ^8^ University of South Australia, Quality Use of Medicines and Pharmacy Research Centre, UniSA Clinical and Health Sciences Adelaide SA Australia; ^9^ Australian Institute of Health Innovation Macquarie University Macquarie Park NSW Australia; ^10^ College of Education, Psychology and Social Work Flinders University Adelaide SA Australia; ^11^ Australian Nursing and Midwifery Federation (SA Branch), Adelaide SA Australia; ^12^ Adelaide Geriatrics Training and Research with Aged Care (GTRAC) Centre, School of Medicine, Faculty of Health and Medical Sciences University of Adelaide Adelaide SA Australia; ^13^ Aged and Extended Care Services, The Queen Elizabeth Hospital, Central Adelaide Local Health Network SA Australia; ^14^ Department of General Practice University of Melbourne Parkville VIC Australia; ^15^ Embedded Health Solutions Melbourne VIC Australia; ^16^ School of Nursing and Midwifery Monash University Clayton VIC Australia

**Keywords:** health care, health services for the aged, home care, medicines, monitoring, nursing homes, patient safety

## Abstract

People accessing aged care services are increasingly older and often experience multimorbidity and polypharmacy, which puts them at risk of medication‐related harm. Quality indicators (QIs) can assist with monitoring, benchmarking and informing initiatives to reduce medication‐related harm. This systematic review aimed to identify and summarize QIs that assess the safe and effective use of medications in long‐term care services.

Bibliographic databases and grey literature were searched to identify relevant QIs. Eligible publications were in English and described the development, application and/or validation of QIs in long‐term care facilities or in‐home aged care services. QI information, including their development and settings, were extracted. All QIs were classified according to 3 validated classification systems and grouped by themes constructed from the review.

From the 62 academic articles and 16 grey literature documents included, 53 QI sets were extracted, which comprised 442 individual QIs and 18 potentially inappropriate medication lists were identified. Most (80%, *n =* 354) QIs were process indicators. About 1/4 (26%, *n =* 115) were medication‐specific QIs focusing mainly on prevalence of use and dosing, with similar numbers for infection prevention and control (25%, *n =* 112). A smaller proportion (7%, *n =* 32) of QIs encompassed person‐centred measures such as resident involvement in medication‐related decisions.

This comprehensive overview of contemporary QIs to monitor medication safety and effectiveness across long‐term care services can help clinicians, aged care providers and policy makers to identify important measures to employ in aged care settings to monitor and influence care improvements.

## 1 INTRODUCTION

Increasing longevity and population growth has led to a rapidly growing number of older adults requiring long‐term care services.^1^, ^2^ Across Organization for Economic Co‐operation and Development countries, in 2021 an average of 11.5% of adults aged 65 years and over received long‐term care in dedicated facilities or at home.^3^ Long‐term care facilities (LTCFs), also known as nursing homes, aged care homes or residential aged care facilities, are facilities where older adults with high care needs reside, often in the final months or years of their life.^4^ In‐home aged care services (HC) refer to a broad range of aged care services that are provided in people's homes, such as specialized clinical services, personal care and services to support activities of daily living.^5^ On average, Organization for Economic Co‐operation and Development countries allocate 1.8% of their gross domestic product to long‐term care^3^ with a substantial increase in spending on both LTCFs and HC projected in the coming years.^6^ The LTCF and HC populations commonly experience frailty and multimorbidity, leading to polypharmacy and complex medication regimens, making this population particularly vulnerable to medication‐induced harm.^7^, ^8^ Use of high‐risk medications, such as psychotropics, anticoagulants and opioids, as well as potentially inappropriate medications (PIMs), where the risk associated with use may outweigh the benefits, are highly prevalent among older individuals accessing LTCFs^8^, ^9^ and HC.^7^, ^10^ Over 95% of residents in LTCFs experience at least 1 medication‐related problem,^11^ which is a major contributor to avoidable adverse drug events and hospitalizations.^12^, ^13^ In Australia alone, it is estimated that approximately a quarter of all unplanned hospitalizations are medication‐related, with the older population most affected.^14^

The reduction of medication‐related harm is an international health priority,^15^ and aged care services are an important initial target.^16^ Hence, there is a growing interest in improving medication management systems in aged care services and in monitoring quality improvement efforts. Current strategies include provision of multidisciplinary collaborative services involving pharmacists, such as comprehensive medication reviews, deprescribing initiatives and educational interventions.^11^, ^17^, ^18^, ^19^, ^20^ Australia has recently implemented an Aged Care Onsite Pharmacist model in LTCFs^16^ that enables pharmacists to provide individual resident level clinical services, clinical governance and educational services to support safe and effective medication use among residents.^11^, ^21^ Despite this, there has been little focus on the routine evaluation of the broad spectrum of medication management, including the evaluation of pharmacists' services, in LTCF and HC.

Quality monitoring programmes and their associated quality indicators (QIs) are commonly used to assess care quality and monitor changes at the local or population level. QIs typically focus on measuring the structures, processes or outcomes of care.^22^ QIs further allow for the comparison and benchmarking of service quality across institutions to identify areas for improvement and to evaluate subsequent interventions over time. A variety of medication‐related QIs are monitored at the population level, such as the USA's Centers for Medicare and Medicaid Services minimum data set for resident assessment,^23^ or the Australian National Mandatory Quality Indicators,^24^ which include 2 medication management QIs (prevalence of antipsychotic use and polypharmacy). An increasing amount of literature has been dedicated to the development of QIs for the assessment of geriatric pharmacotherapy, including screening using PIM lists,^25^, ^26^ that can serve as tools to evaluate the appropriateness of medication use among older adults. However, developed QIs often focus on 1 specific aspect of geriatric pharmacotherapy (e.g., polypharmacy) rather than an overall evaluation of all medication‐related activities (e.g. medication reviews, pharmacist's services) in LTCFs and HC, limiting their clinical utility.

Providing a comprehensive and timely overview of QIs and PIM lists that cover a broad range of medications, medication‐related activities and pharmacist services in LTCF and HC can be used to monitor and drive care improvements to reduce medication‐related harm and improve quality of care in these settings. The objective of this systematic review was to identify, examine and summarize existing QIs, including PIM lists, from academic and grey literature, that assess the structure, processes and outcomes related to the safe and effective use of medications in LTCFs and HC.

## 2 METHODS

### 2.1 Search strategy

The Preferred Reporting Items for Systematic reviews and Meta‐analysis (PRISMA) guidelines were followed for this systematic review. The protocol was preregistered on PROSPERO (CRD42023442537). The academic literature search was conducted on 9 August 2023 across the following 4 databases: Ovid MEDLINE, Ovid EMBASE, Ovid PsycINFO and CINAHL EBSCOhost. The search was restricted to English and to publication dates from January 2013 onwards, to provide an update to the review conducted by Hillen *et al*.^27^ in 2013 that synthesized medication‐related QIs for LTCFs. A combination of keywords and MeSH terms that relate to the safe and effective use of medications (also known as quality use of medicines) or pharmacists' services, QIs and LTCFs or HC services were used (see Appendix Table S1 for full search strategy). Additionally, a grey literature search was conducted using Google (search terms: quality indicator, quality standards, quality measures, performance measures and performance indicators AND long‐term care, nursing home, residential aged care, community care), restricted to the first 10 pages. Potentially relevant websites of organizations known to the authors or found during the process of the academic literature review were searched (see Appendix Table S2). Additionally, the list of identified QIs from a large scoping review of QIs used in population‐based surveillance programmes to monitor the quality and safety of care in older people across LTCF, HC, palliative care and care transitions was reviewed to identify additional QIs that met our inclusion criteria.^28^

### 2.2 Eligibility criteria

For the academic literature, original and peer‐reviewed studies that examined the development, testing or implementation of QIs to assess the structure, processes or outcomes related to the safe and effective use of medications among older adults (≥65 or ≥50 years for Indigenous peoples) accessing either LTCFs or HC services were included. QIs specifically relating to safe and effective use of medications (e.g. treatment decisions, medication monitoring) and medication management services (e.g. medication reviews, pharmacist services) were included. PIM lists were included if they were either developed to assess medication use quality in LTCFs or HC or used to evaluate the quality of medication related care in LTCFs or HC. Studies were excluded if the QIs only targeted populations with care needs outside the scope of the aged care population (e.g. HIV, obstetrics) or with acute disorders usually managed in hospitals (e.g. myocardial infarction). Additionally, measures focusing on hospital settings, emergency care and surgery, hospital at home, psychiatric facilities, dentistry and inpatient palliative care units (hospices) were excluded. Conference abstracts, editorials, letters, case reports, case series, protocols, dissertations and narrative reviews were also excluded. Systematic reviews that only amalgamated pre‐existing indicators were excluded but screened for included articles that met the date, population, setting and content criteria. Similarly, articles that only discussed the application or adaption of an existing QI were excluded but were screened to identify the original indicator set. For the grey literature review, policy documents, manuals, clinical practice guidelines and documents from government departments or health organizations were included. QIs that were not publicly available (i.e., behind a paywall), or not in English were excluded.

### 2.3 Study selection

**FIGURE 1:**
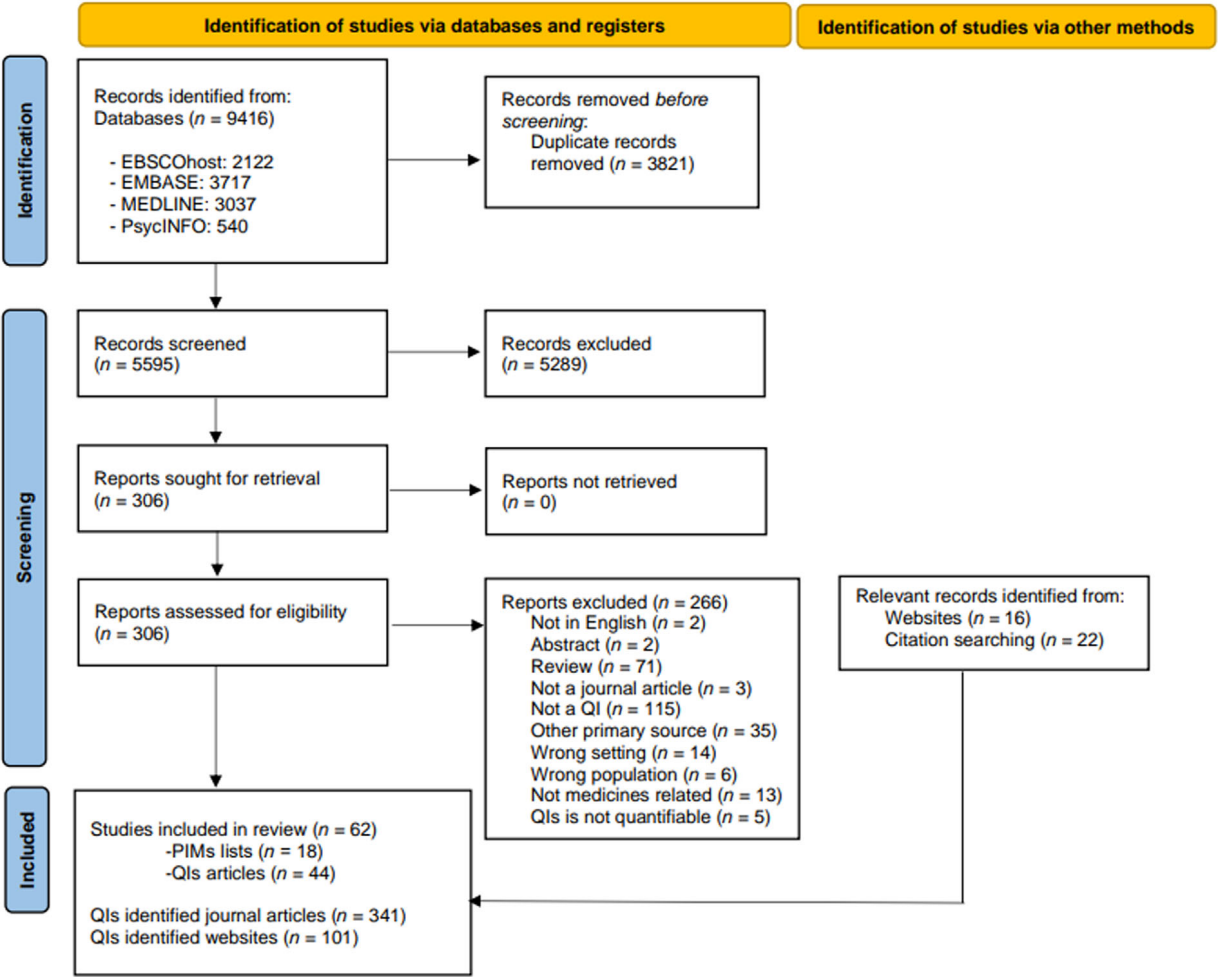
PRISMA flow diagram for selection of studies and indicators. PIM, potentially inappropriate medication; QI, quality indicator.

The article selection and screening process is shown in the PRISMA flow diagram (Figure 1). Identified references were extracted into a reference management system, where duplicates were removed and subsequently uploaded into the systematic review management software Covidence (Melbourne, 2023). Two reviewers (D.S.G. and A.C. or J.Stas.) independently screened all abstracts and titles for eligibility. Subsequently, all full texts of relevant articles were independently examined against the eligibility criteria by the same reviewers. Any disagreements between the reviewers were resolved through discussion and, if necessary, through consultation with a third researcher (J.K.S.).

### 2.4 Data extraction and synthesis

All data were extracted and categorized by 1 researcher (D.S.G.) and verified by a second (A.C.), with any discrepancies resolved by discussion. In line with past systematic reviews into QIs,^29^, ^30^ the following information was extracted for each QI set: source and name of the QI set (if applicable), publication year, country in which the QI set was developed or used, aim and development of the QI set including setting (i.e., LTCF or HC), total number of QIs in each set and whether they are used on a population‐level (i.e. QI programs that are publicly reported on a national, state or regional level). The definition of each QI (including the numerator and denominator used to calculate the QI result, if available) and, if applicable, its exclusion criteria, risk adjustments, stratification and performance target were extracted. Duplicated QIs from the same author group or indicator programme were not extracted. QIs that were the same across different QI lists from different sources were extracted and flagged as duplicates (see Table S3).

To capture the multidimensional components of the safe and effective use of medications, multiple classification systems were used to classify the QIs. The following 3 classification systems were used to categorize and synthesize individual QIs: (i) indicator type (i.e., structure, process, outcome) using Donabedian's framework^31^; (ii) the 9 causes of drug‐related problems (c‐DRP) classification system^32^ (i.e., drug selection, drug form, dose selection, treatment duration, drug use process, logistics, monitoring, adverse drug reactions and other); and (iii) the 9‐item Medication Management Pathway^33^ classification system, comprising: decision to prescribe; record medication order; review of medication order, referring to the verification of information and clinical appropriateness consideration before medication is dispensed; issue (i.e., dispensing) of medication; provision of medication information; distribution and storage of medication; administration of medication; monitor for response; and transfer of verified information. If the QIs did not fall in any of the above categories, they were classified as other.

In addition, we categorized all QIs based into 8 themes, derived from: (i) reviewing all QIs and (ii) the identification of priority areas in aged care and pharmacy‐related policies to ascertain the key areas covered by QIs^24^, ^34^: (i) infection prevention and control, including vaccination rates and antimicrobial stewardship; (ii) medication‐specific indicators; (iii) comprehensive medication reviews (overall and situation‐specific) and medication reconciliation; (iv) medication incidents, including adverse drug events; (v) medication risk assessment, prevention and monitoring strategies; (vi) person‐centred measures, such as resident involvement in medication‐related decisions; (vii) pharmacist services, including interdisciplinary collaboration and provision of staff education; and (viii) palliative and end‐of‐life care.

## 3 RESULTS

### 3.1 Study selection

A total of 9416 articles were extracted, from which 3821 duplicates were removed, leading to 5595 articles included in the initial title and abstract screening (Figure 1). Of those, 306 full text articles were assessed for eligibility and 266 articles were excluded (Figure 1). An additional 22 journal articles, identified via citation and website searching, were included, leading to 62 included articles, containing 37 individual QI sets and 18 PIMs lists. The grey literature search identified 16 relevant websites containing 16 QI sets. As a result, a total of 53 QI sets (Table 1), comprising 442 individual QIs (Appendix Table S3) were included in this review.

**TABLE 1:** Overview of included quality indicator (QI) sets organized by academic and grey literature.

QI set	Author, year (name of QI set if available)	Country	Aim of the QI set	Development	Number of QIs				Setting		Applied at population level
					T	S	P	O	LTCF	HC	
**Academic literature**											
1	Asquier‐Khati *et al*. (2023)^35^	France	Antimicrobial stewardship QIs for local and national monitoring	RAND‐modified Delphi	22	0	22	0	X		
2	Ayton *et al*. (2020)^36^	Australia	Clinical QIs for an Australian dementia registry	Literature review, Delphi	1	0	1	0	X	X	
3	Cations *et al*. (2021)^37^	Australia	National surveillance of clinical QIs for dementia care	Selected literature of international Delphi studies	3	0	3	0	X	X	
4	De Schreye *et al*. (2017)^38^	Belgium	Population based end‐of‐life care measures in people with Alzheimer's disease	Systematic literature review, modified RAND/UCLA appropriateness method	10	0	10	0	X	X	
5	De Souto Barreto *et al*. (2013)^39^	France	Quality of health care provided in nursing homes	Based on recommendation of health authorities in France	4	0	4	0	X		
6	Doran *et al*. (2014)^40^	Canada	Identification of serious events in home care	Delphi	1	0	0	1		X	
7	Favez *et al*. (2020)^41^	Switzerland	Swiss national nursing home QIs	Literature review, expert consensus via RAND/UCLA method, usability assessment	1	0	1	0	X		X
8	Frijters *et al*. (2013)^42^ interRAI	Netherlands	QIs for long‐term care facilities in 8 countries (SHELTER project)	Based on interRAI LTCF assessment	3	0	3	0	X		X
9	Fujita *et al*. (2021)^25^	Japan	QIs for pharmacist home visit services	Systematic literature review, national guidelines, health care professional workshop, expert consensus testing (RAND/UCLA appropriateness method), field evaluation, interviews	44	1	36	7		X	
10	Hertig *et al*. (2016)^43^	USA	Medication safety measurements for long‐term care pharmacy	Failure models effect analysis based on flowchart data, expert panel (consisting of pharmacists), consensus panel	6	0	0	6	X		
11	Hibbert *et al*. (2022)^44^	Australia	Clinical QIs for common residential aged care conditions and processes of care	Systematic search of international clinical practice guidelines, modified Delphi approach, ratification via expert panel	65	0	65	0	X		
12	Inacio *et al*. (2020)^45^ and Caughey *et al*. (2022)^46^	Australia	Quality and safety indicators for long‐term care that are suitable for registry data	Literature review and expert engagement	6	0	5	1	X	X	X
13	Kalisch Ellett *et al*. (2021)^47^ and Caughey *et al*. (2014)^48^	Australia	Medication‐related hospitalization indicators	Based on treatment recommendations from evidence‐based clinical guidelines, validated by expert panel in population‐based sample	21	0	0	21	X		
14	Katz *et al*. (2022)^49^	USA	Antimicrobial stewardship indicators for long‐term care	Not specified	8	4	4	0	X		
15	Kosari *et al*. (2021)^50^	Australia	Nursing home medication QIs	Not specified	9	0	6	3	X		
16	Leemans *et al*. (2017)^51^; Leemans *et al*. (2015)^52^; Cohen *et al*. (2021)^53^	Belgian	Indicators for palliative care services	2‐round modified RAND/UCLA panel	2	0	0	2	X	X	
17	Lindskog *et al*. (2015)^54^	Sweden	Population‐based end‐of‐life care quality from the Swedish register of palliative care	Based on Swedish registry of palliative care questionnaire, previous systematic review and expert panel	6	0	6	0	X	X	
18	Mays *et al*. (2018)^55^	USA	QIs for primary care provider engagement in nursing homes	Previous developed indicators from ACOVE 3 QIs (2007), NH QIs (2004), NH Residential Care QIs (2002) and AGS Choosing Wisely (2014) were adapted by 7 international members, international RAND modified Delphi	38	0	38	0	X		
19	McDerby *et al*. (2020)^56^	Australia	Medicines QIs for residential aged care	Not specified	4	0	3	1	X		
20	Nguyen *et al*. (2021)^57^; Nguyen *et al*. (2020)^58^	United Kingdom	Clinical trial antimicrobial stewardship QIs for care homes that are important for stakeholders	Systematic literature review, interviews with key stakeholders, international Delphi	11	0	9	2	X		
21	Pont *et al*. (2018)^59^	Australia	Quality use of medicines in aged care	Not specified	3	0	3	0	X		
22	Prasanna *et al*. (2020)^60^	Sri Lanka	Medication error indicators	Based on published literature and expertise of authors and clinical pharmacists	4	0	0	4	X		
23	Rahja *et al*. (2022)^61^	Australia	Clinical QIs in dementia care	QIs that were considered by the Australian dementia network clinical quality registry	1	0	1	0	X	X	
24	Reichert *et al*. (2018)^62^	Germany	Quality of care indicators	Based on past literature	8	0	7	1	X		
25	Religa *et al*. (2015)^63^	Sweden	Swedish Dementia Registry (SveDem) QIs	Indicators were taken from the Swedish national guidelines for care in cases of dementia	2	0	2	0	X	X	X
26	Sanchez *et al*. (2022)^64^; Sanchez *et al*. (2022)^65^	France	Prescription quality of drugs with anticholinergic properties	Developed in collaboration between the researchers and the medical directors of nursing homes, focus groups	4	0	4	0	X		
27	Schjøtt *et al*. (2019)^66^	Norway	Psychotropic prescribing quality in nursing homes	Based on past academic literature where the indicator was tested with Swedish national data	3	0	3	0	X		
28	Shin *et al*. (2015)^67^	Korea	Quality of care in nursing homes	Taken from the 2013 NH evaluation manual by the Korean National Health Insurance Service	2	0	2	0	X		
29	Simon *et al*. (2021)^68^	France	Appropriateness of antibiotic prescribing in nursing homes	Literature review, structured consensus procedure, adjusted to French context	26	0	26	0	X		
30	Tang *et al*. (2018)^69^	China	QIs for home care in China	Literature review, field study to identify key quality control issues, qualitative interviews with stakeholders, expert panel/consultation, Delphi	4	2	2	0		X	
31	Tate *et al*. (2022)^70^	Canada	QIs for older person's transition in care	Systematic review, Delphi, feasibility review by steering committee	2	1	1	0	X	X	
32	Uittenbroek *et al*. (2016)^71^	Netherlands	Quality of integrated care from the perspective of elderly people	Adapted previous established measures, tested in small sample	1	0	1	0		X	
33	Wagner *et al*. (2020)^72^ interRAI	Switzerland	Inter RAI‐HC Switzerland—QIs for home‐care	Systematic literature search of inter‐RAI QIs, expert panel rounds (RAND/RAM method), focus groups of health care providers/nurses rating clinical appropriateness of QIs	1	0	0	1		X	X
34	Walus *et al*. (2017)^73^	Canada	Utility of acute care clinical pharmacy performance indicators for home care pharmacy services	Original set was developed via modified Delphi for clinical pharmacy service, this study used them on a home care data set to test applicability	3	0	2	1		X	
35	Winblad *et al*. (2017)^74^	Sweden	QIs of care for nursing homes, based on Swedish National Board of Health and Welfare	Developed by the Swedish National Board of Health and Welfare, in collaboration with experts and health professionals	2	0	2	0	X		X
36	Winters *et al*. (2014)^75^	Netherlands	Residential care	Based on the 2012 Dutch national set of Qis	4	0	3	1	X		X
37	Woitha *et al*. (2015)^76^; Woitha *et al*. (2014)^77^	Netherlands	Palliative care QIs	Literature review, modified RAND Delphi, feasibility and reliability assessment across 25 European countries	4	3	1	0	X		
**Grey literature**											
38	Canadian Institute for Health Information (CIHI)^78^ interRAI	Canada	Continuing Care Reporting System, QIs for residential care	Developed by interRAI, an international research network	1	0	1	0	X		X
39	CIHI Home Care Reporting System^79^ interRAI	Canada	QIs for home care	InterRAI	2	0	1	1		X	X
40	Ontario Health^80^	Canada	Long‐term home care performance QIs	Not specified	1	0	1	0		X	X
41	Centers for Medicare and Medical Services (CMS)^23^	USA	Quality measures for nursing homes	Developed, refined and tested since 1990, incorporating end‐users' perspective. Extensively used and tested.	12	0	12	0	X		X
42	CMS^81^	USA	Quality measures for home health	Developed, refined and tested since 1990, incorporating end‐users' perspective. Extensively used and tested.	13	0	8	5		X	X
43	National Institute for Health and Care Excellence (NICE)^82^	UK	Medicines management in care homes; antimicrobial stewardship; dementia care	Topics are referred to NICE via different organization, literature search, impact and cost analysis, consultation of committee of professionals, and families, guideline consultation	26	9	13	4	X	X	
44	Australian Government Department of Health and Aged Care^16^	Australia	National aged care mandatory QIs	Evidence‐based literature review, national stakeholder consultation, expert consultations, testing with providers and stakeholders, trial in residential aged care services	2	0	2	0	X		X
45	Australian Commission on Safety and Quality on Health Care report^16^	Australia	QIs for quality use of medicines in aged Care	Stakeholder engagement	18	0	15	3	X		
46	Department of Health Victoria^83^	Australia	QIs for residential aged care services	Literature review, expert consultation	1	0	1	0	X		X
47	Victorian Healthcare Associated Infection Surveillance data (VICNISS)^84^	Australia	Infection surveillance indicators for aged care	Not available	5	0	4	1	X		X
48	Australian Institute of Health and Welfare^85^	Australia	QIs for national reporting	Consultation with stakeholders, review of grey literature, advice from the ACSQHC, discussed with key stakeholders and refined	2	0	1	1	X		
49	National Healthcare Safety Network (NHSN)^86^	USA	QIs from the centres for disease control and prevention for long‐term care	Based on common issues faced in LTC, collaboration with key stakeholders, continuing testing and validation of QIs via the Minimum Data Set	4	0	3	1	X		X
50	SveDem‐ Swedish Dementia registry^63^	Sweden	Dementia care QIs	Collaboration with key stakeholders, and based on an evidence‐based framework for dementia care, validation and continuous monitoring and evaluation	2	0	2	0	X	X	X
51	THL Finnish Institute for Health and Welfare interRAI^87^	Finland	Residential aged care QIs	InterRAI	5	0	5	0	X		X
52	Icelandic Minimum Data Set Quality Indicators interRAI^88^, ^89^	Iceland	Residential aged care QIs	InterRAI	5	0	5	0	X		X
53	National Centre for Antimicrobial Stewardship (NCAS; partner with VICNISS)^90^	Australia	Antimicrobial prescribing	Ongoing collaborative partnership between the National Centre for Antimicrobial Stewardship the Australian Commission on Safety and Quality in Health Care and the Australian Government Department of Health and Aged Care.	2	0	2	0	X		X

Abbreviations: ACSQHC, Australian Commission on Safety and Quality in Health Care; ACOVE, acessing care of vulnerable elders; AGS, American geriatrics society; HC, home care; interRAI, international resident assessment instrument; LTCF, long‐term care facilities; NH, nursing home; NICE, national institute for health and care excellence; O, outcome; P, process; S, structure; T, total.

### 3.2 QI set characteristics

The 53 included QI sets and QIs contained in each set are summarized in Table 1. Nearly half (*n =* 22, 42%) of the QI sets originated from Europe across 10 countries, followed by Australia (*n =* 15, 28%). Another 12 (23%) indicator sets were from North America (USA *n =* 6, Canada *n =* 6) and 4 (8%) were from Asia, including indicators from Japan, China, Korea and Sri Lanka. Thirty‐five QI sets (66%) contained indicators for LTCFs, 7 (13%) for HC and 11 (21%) targeted both LTCFs and HC. Almost half of the QI sets (*n =* 22, 42%) aimed to assess general aged care service quality, 7 focused (13%) on infection prevention and control, 5 (9%) on dementia care, 4 (8%) focused on palliative or end‐of‐life care and 1 (2%) focused on the transitions of care. Seven (13%) QI sets specifically focused on the safe and effective use of medication, 3 (6%) on adverse drug events or medication errors, 2 (4%) on pharmacists' services and 1 (2%) QI set focused on person‐centred quality. Additionally, the National Institute for Health and Care Excellence website contained multiple QI sets, focusing on medication management, antimicrobial stewardship and dementia care. These were included but summarized as 1 QI set. The QI sets containing the highest number of QIs related to the safe and effective use of medications, were extracted from Hibbert *et al*.^44^ (65 QIs, focused on LTCFs), followed by Fujita *et al*.,^25^ who developed QIs for HC pharmacist services (44 QIs).

Of the 53 QI sets, 38% (*n =* 20) had been applied at a population‐level, of which 6 (11%) were based on the interRAI^91^ Adult and Elderly Care instruments (Table 1). Of the 33 (63%) QI sets not used at the population level, 15 (28%) were developed via a structured expert consensus process (e.g. Delphi) and 7 (13%) were developed based on existing literature or other recommendations, with no further details provided (Table 1).

### 3.3 Individual QI characteristics

**FIGURE 2:**
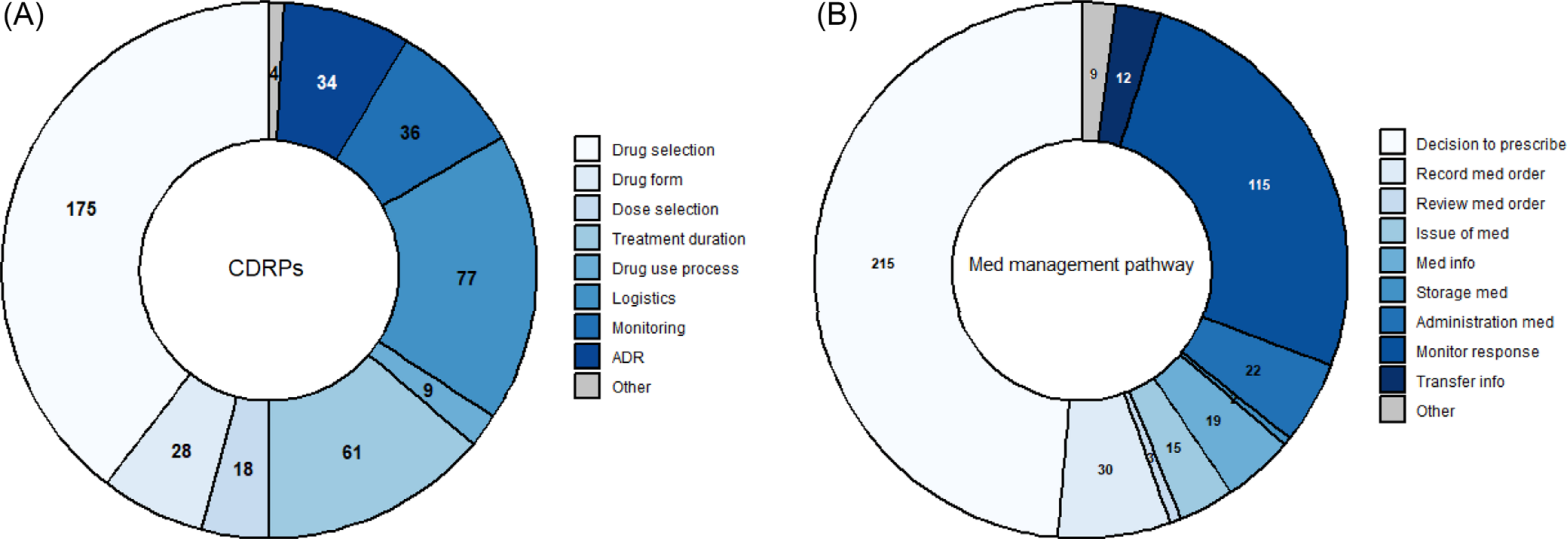
Number of individual quality indicators (QIs) classified by: (A) the causes of drug related problems (CDRPs) classification system and (B) the steps of the medication management pathway cycle. ADR, adverse drug reaction; med, medication.

The 442 individual QIs, including their categorizations, are outlined in the Appendix (Table S3). We identified a total of 11 duplicates, leading to a total of 431 unique QIs. Seventeen percent (*n =* 75) of all QIs had been applied on a population level. Of the remaining QIs 65% (*n =* 239 out of 367) were developed via a structured expert census process, as outlined in Table 2. The vast majority of the QIs were for LTCFs (*n =* 367, 83%). Most of the QIs (80%) were process indicators, followed by outcome indicators (15%; see Table 2). Less than 5% were structural indicators, of which none had been applied at a population level. Over half of the identified QIs lacked a clearly defined numerator and denominator (*n =* 258, 59%), most of which originated from academic literature (*n =* 224/258, 87%). Categorization by c‐DRP^32^ showed that the most common types of QIs focused on drug selection (40%, *n =* 175), followed by logistics (17%, *n =* 77) and treatment duration (14%, *n =* 61; Figure 2). Based on the medication management pathway, approximately half (49%, *n =* 215) of the QIs related to the decision to prescribe, followed by monitoring for response to medication (26%, *n =* 115). A small number (<1%, *n =* 3) of QIs related to the review of a medication order, medication storage (<1%, *n =* 2) or other (2%, *n =* 9).

**TABLE 2:** Number of quality indicators (QIs) developed for each setting and applied at the population‐level, organized by Donabedian framework and emerged themes.

*N* (%)	Total number of QIs	Setting			Population‐level QIs	
		LTCF	HC	Both	Yes	No
**Donabedian**						
Structure	20 (4.5)	13 (4.0)	6 (7.5)	1 (2.5)	0 (0)	20 (5.5)
Process	354 (80.1)	268 (83.2)	57 (71.3)	29 (72.5)	64 (85.3)	290 (79.0)
Outcome	68 (15.4)	41 (12.7)	17 (21.3)	10 (25.0)	11 (14.7)	57 (15.5)
**Themes**						
Infection prevention and control	112 (25.3)	100 (31.1)	12 (15.0)	0 (0)	25 (33.3)	87 (23.7)
Medication specific	115 (26.0)	90 (28.0)	13 (16.3)	12 (30.0)	32 (42.7)	83 (22.6)
Medication review and reconciliation	40 (9.1)	32 (9.9)	5 (6.3)	3 (7.5)	6 (8.0)	34 (9.3)
Medication incidents	39 (8.8)	30 (9.3)	2 (2.5)	7 (17.5)	3 (4.0)	36 (9.8)
Palliative and end‐of‐life care	22 (5.0)	12 (3.7)	0 (0)	10 (25.0)	0 (0)	22 (6.0)
Person‐centred	32 (7.2)	20 (6.2)	8 (10.0)	4 (10.0)	9 (12.0)	23 (6.3)
Pharmacists' services*	32 (7.2)	3 (0.9)	29 (36.3)	0 (0)	0 (0)	32 (8.7)
Medication risk assessment, prevention and monitoring	50 (11.3)	35 (10.9)	11 (13.8)	4 (10.0)	0 (0)	50 (13.6)
**Total**	442 (100)	322 (72.9)	80 (18.1)	40 (9.1)	75 (17.0)	367 (83.0)

*Note*: Population level refers to indicators that have been implemented on national, state or regional level.

Abbreviations: HC, home care; LTCF, long‐term care facility.

*QIs about medication reviews and reconciliation were categorized separately.

Of the 8 themes identified from the QIs, approximately a quarter (26%, *n =* 115) of the QIs were medication‐specific indicators, mainly relating to psychotropic and analgesic use, as well as polypharmacy. Another quarter (25%, *n =* 112) of the QIs focused on infection prevention and control, and mainly comprised influenza vaccination rates and antimicrobial use (Table 2). Seven percent of the QIs focused on a person‐centred measure, such as residents' involvement in medication‐related decisions. Pharmacist services were also targeted by 32 QIs; however, only 3 of these QIs were developed for LTCFs. The following themes had not been implemented in QIs at a population level: (i) medication risk assessment, prevention and monitoring; (ii) pharmacists' services; and (iii) palliative and end‐of‐life care (see Table 2). Palliative and end‐of‐life care QIs primarily focused on anticipatory medications and deprescribing.

### 3.4 PIM lists

This systematic review identified 18 different PIM lists that have been developed or used to evaluate the appropriateness of medication use among older adults in LTCFs or HC. Three PIM lists were specifically developed for LTCFs and 1 was developed for HC (Table S4). The majority of PIM lists were developed using the Delphi method (Table S4). Of the 4 most commonly used PIM lists (i.e., Beers criteria,^92^ STOPP Frail V2,^93^ EU‐7^94^ and NORGEP‐NH^95^) identified in this review, the following were the most common medication classes: antidepressants, antihypertensives, nonsteroidal anti‐inflammatories, antipsychotics, antithrombotics, benzodiazepines, bisphosphonates, corticosteroids for systemic use, glucose lowering medications, lipid modifying agents, opioids, proton‐pump inhibitors, and medications for urinary frequency and incontinence.

## 4 DISCUSSION

This systematic review identified 53 QI sets with 442 individual QIs, as well as 18 PIM lists, to assess quality use of medications among older adults in long‐term care settings. Nearly 40% of the QI sets were used at the population‐level and the majority of the individual QIs were developed via a structured expert consensus. The identified indicators are a valuable starting place for monitoring, benchmarking and evaluating medication‐related care in long‐term care services to minimize medication‐related harm. However, there is still room for improvement in defining numerators and denominators and developing risk adjustments for some indicators before they can be applied in LTCFs or HC.

The identified indicators target a broad spectrum of medication‐related themes, including medication‐specific measures, infection prevention and control and person‐centred metrics, that are important for safe and effective medication use in long‐term care services.^96^ However, QIs have only been applied at the population level for a subset of these themes, with 76% of population level QIs focusing on specific medication classes or infection prevention and control (including antimicrobial stewardship). None of the QIs applied at a population level covered palliative and end‐of‐life care, pharmacists' services or medication risk assessment, prevention and monitoring. In addition, few QIs were identified that measured the outcomes of medication‐related care quality, with only 7 QIs developed for population‐level monitoring within LTCFs. These findings are in line with the previous systematic review conducted by Hillen *et al*.^27^ that identified a lack of QIs addressing medication use in residents of LTCFs with limited life expectancy, as well as limited outcome focused QIs. Notably, all medication‐specific QIs identified by Hillen *et al*. were also covered within the present study, except for QIs examining the *triple whammy* ^97^ combination.

Differences between the type and focus of QIs identified in the academic literature and those applied at the population level may indicate either an implementation gap or feasibility concerns. It is common to see a delay between published findings and adoption into practice.^98^ QIs implemented at the population level are usually well defined and have undergone comprehensive validation and testing within their target population.^99^ However, we found that many QIs identified in the academic literature lacked clear definitions for both the numerator and denominator, making reproducibility and implementation difficult. Information on how to collect data for these QIs was often absent, particularly for person‐centred measures, where it was sometimes unclear whether data should be collected from care recipients or providers. In addition, few QIs from the published literature outlined exclusion criteria and risk adjustments, which are particularly important given the characteristics of the individuals accessing care can influence QIs independent of the quality of care provided.^29^ Hence, future QI developers should provide clear definitions with numerators, denominators and address data collection and potential for risk adjustments to increase the usability of an indicator. For example, resident characteristics, such as cognitive and functional impairment and resulting care needs can vary across LTCFs, which may lead to differences in medication use and resident outcomes and without this individual level information is difficult to account for when comparing facilities. Hence, further work is needed to refine the definitions of many QIs identified by this review if they were to be used for quality assessment and benchmarking across different care settings and regions. The underrepresentation of outcome‐focused QIs used at the population level may be partly because health outcomes are often influenced by multiple factors, some of which are beyond the control of individual healthcare providers.^30^ Additionally, there may be barriers to monitoring client outcomes over time. This suggests a need for improved availability and access to linked health and aged care datasets to support routine monitoring of key health outcomes, such as medication incidents and hospitalizations, to inform aged care practice and policy decisions.

One‐quarter of the identified QIs were medication‐specific, focusing on the prevalence of use of high‐risk drug classes or composite measures such as polypharmacy, sedative load, anticholinergic burden or PIMs. Use of PIM lists, such as the Beers^92^ criteria or NORGEP‐NH,^95^ can help to identify individuals who may benefit from a comprehensive medication review. However, as described in a recent umbrella review by Anlay *et al*.,^100^ PIM lists generally contain many different medications, sometimes over 100. There is considerable variation in uptake of electronic medication management systems in LTCFs in Australia and internationally.^101^ Therefore, QIs focused on PIM lists may be time‐consuming and burdensome to implement at the population level. Use of paper‐based medication administration charts can also present barriers for PIM data collection and reporting. Importantly, our review identified individual process indicators for the majority of medication classes commonly included in PIM lists, except for antihypertensives and bisphosphonates.

The current review identified a gap in QIs that relate to pharmacists' services in HC and LTCFs. Classification by the c‐DRP classification system and the Medication Management Pathway found the identified QIs and PIM lists had sufficient coverage of interventions amenable to change through clinical pharmacy practice, such as the decision to prescribe and medication selection.^11^ However, other areas such as dispensing, administration, medication‐related risk assessment and monitoring were less well covered. It may be that dispensing QIs are not covered as community pharmacists are not typically colocated with an LTCF or HC provider. While the current review identified 32 QIs that focused specifically on pharmacists' services, the majority of these indicators were developed for HC.^25^ Few QIs focused on key areas such as clinical governance, education and interprofessional communication and collaboration that are essential for the safe and effective use of medications.^20^ An additional 40 QIs focused on medication review or reconciliation, which are commonly performed by pharmacists in collaboration with other health care professionals. Most of these, however, were developed for HC and none have been applied at a population level. Pharmacist practice models in LTCFs have emerged in multiple countries, such as England, the USA and Australia.^11^ However, the extent to which the pharmacists are integrated into LTCFs varies, which can impact interprofessional communications.^11^ Australia has recently commenced a model of embedding pharmacists onsite within LTCFs to support the safe and effective use of medications and person‐centred care to improve resident outcomes.^102^

The QIs identified by this review will be further assessed as part of the PHarmacists Actioning Rational Use of Medicines in Aged Care (PHARMA‐Care) programme. The PHARMA‐Care programme is developing, implementing and evaluating a QI framework for monitoring medication management and pharmacist services suitable for national scaling across Australian LTCFs. A sub‐selection of QIs relevant for the Australian context will be rated by an expert panel for importance, feasibility and amenability to change via a Delphi process, and barriers and enablers will be identified via qualitative interviews.

### 4.1 Strengths and limitations

Strengths of this review include the inclusion of QIs related to LTCFs and HC for older adults. We followed the PRISMA guidelines and applied a comprehensive search strategy developed with the assistance of academic librarians. All abstracts, full texts and grey literature were screened by 2 reviewers independently and all data extraction and QI categorizations were checked by a second reviewer to minimize errors. Importantly, we conducted a search of websites and policy documents, to include newly developed QIs and QIs commissioned by governments. Categorizing all QIs into 3 validated classification systems (i.e., Donabedian framework, c‐DRPs and the Medication Management Pathway) and 8 themes constructed from a qualitative analysis of the QIs, helped identify gaps in the currently available QIs and provides a direction for future research.

Study limitations included that the search was limited to QIs freely accessible and in English. It is possible that QIs relevant to culturally and linguistically diverse populations, where for instance family care or the use of traditional and complementary medications may be more common, were under‐ascertained.^103^ Additionally, the feasibility and usefulness of QIs is dependent on the healthcare infrastructure and systems in place, which can vary across countries.^30^ Second, while we screened QIs for concepts related to the safe and effective use of medications in aged care settings, we did not capture more general QIs concerning overall quality of care, resident experience and satisfaction, or QIs from other broader health care settings. While we screened relevant documents and websites to determine whether a QI had been subsequently applied at a population level, the possibility of under‐ascertainment remains. In addition, while information on the development of QI sets and application at the population level, provides some information about the quality of the QIs, an in‐depth assessment of their quality (e.g., importance, scientific acceptability, feasibility and usefulness) was outside the scope of this review as the psychometric properties of LTCF QIs are the intended focus of another ongoing systematic review.^104^ Further research could also consider how the performance of QIs applied on a population level has changed over time and whether the QIs have led to demonstrated changes in health outcomes.

## 5 CONCLUSIONS

This systematic review identified 442 QIs focusing on the quality use of medications that have been developed or applied to assess the quality of care in LTCFs and/or HC, since 2013. Numerous PIM lists have been developed to monitor and guide the use of high‐risk medications in older populations, including within LTCFs and HC services, to prevent adverse drug events. This review identified medication‐specific indicators as well as various QIs relating to medication management, including palliative care measures. Our review provides a wide range of QIs that can be used to guide policy makers and clinicians to assess, monitor and ultimately improve medication management in long‐term care services, as well as identifying gaps in QI development and implementation that can be addressed by future research.

## AUTHOR CONTRIBUTIONS

J.K.S., S.E.J., D.G., G.E.C., A.C.S. and G.M.P. were members of the steering committee overseeing this project. D.S.G., G.E.C., J.B.H., S.E.J., P.D.H. and J.K.S. were members of the working group for this study. J.K.S. conceptualized the study. All authors contributed to the study design. D.S.G. drafted the study protocol and registered it on PROSPERO after review by all authors. D.S.G. ran the literature search. D.S.G., A.H.C., J.S. and G.H.Y. screened the search results and J.K.S. was the third reviewer for consultation. D.S.G., A.H.C. and G.H.Y. extracted the data, and D.S.G., A.H.C. and J.K.S. participated in data analysis and/or interpretation. D.S.G. and J.K.S. drafted the manuscript, and all authors critically revised the initial manuscript draft for important intellectual content. All authors read and approved the final manuscript.

## CONFLICT OF INTEREST STATEMENT

J.K.S. is a nonexecutive director of Southern Cross Care SA, NT, VIC (aged care provider organization). The other authors declare no perceived or actual conflict of interest.

## Supporting information


**TABLE S1** Search strategy for academic literature (Ovid Medline).
**TABLE S2** Institution websites searched for quality indicators.

**TABLE S3** List of quality indicators for the safe and effective use of medications in long‐term care settings.

**TABLE S4** Overview of potentially inappropriate medication (PIM) lists (*n =* 18) that were developed for LTCF or HC, or applied in these populations.

## Data Availability

The data that support the findings of this systematic review are available in the supporting information of this article.
