# Supplementary material for: Quality indicators for safe and effective use of medications in long‐term care settings: A systematic review
Source: Br J Clin Pharmacol. 2025 Aug 18;91(11):3054–69. doi: 10.1002/bcp.70242 (PMC12569564; doi:10.1002/bcp.70242)
Supplement: Supplementary file 1 — TABLE S1 Search strategy for academic literature (Ovid Medline). TABLE S2 Institution websites searched for quality indicators. [file BCP-91-3054-s002.docx]

**Supplementary Table S1:** Search strategy for academic literature (Ovid Medline)

| **#** | **Query** | **Results from 9 Aug 2023** |
| --- | --- | --- |
| 1 | "Quality Indicators, Health Care"/ or "Quality of Health Care"/ or Practice Guidelines as Topic/ or "Potentially Inappropriate Medication List"/ or "Outcome and Process Assessment, Health Care"/ or "Outcome Assessment, Health Care"/ or "Process Assessment, Health Care"/ or Benchmarking/ or Guideline Adherence/ | 354,929 |
| 2 | ("best practice analysis" or benchmark* or donabedian or "health metric*").ti,ab,kf. | 64,604 |
| 3 | ((outcome* or standard) adj2 (evaluat* or assess* or measur* or indicator*)).ti,ab,kf. | 463,411 |
| 4 | ((process* or performance or program* or regulatory or structure or application*) adj2 (evaluat* or assess* or measur* or indicator*)).ti,ab,kf. | 218,804 |
| 5 | (quality adj2 (care or healthcare or evaluat* or assurance or indicator* or assess* or measur* or improv* or criteri* or protocol or standard*)).ti,ab,kf. | 399,546 |
| 6 | (guideline* or consensus or QUM or RUM or HMR or RMMR).ti,ab,kf. | 660,474 |
| 7 | ((clinical or safety or prescribing or prevent*) adj indicator*).ti,ab,kf. | 6,625 |
| 8 | or/1-7 | 1,859,484 |
| 9 | ("quality use of medicine*" or "rational use of medicine*").ti,ab,kf. | 646 |
| 10 | Medication Therapy Management/ or Nonprescription drugs/ or Prescription Drugs/ or Psychotropic Drugs/ or "Drug Therapy, Combination"/ or Drug Tapering/ or Deprescriptions/ or Drug Prescriptions/ or Drug Information Services/ or Drug Interactions/ or Drug Dosage Calculations/ or Inappropriate Prescribing/ or Polypharmacy/ or Medication Errors/ | 347,106 |
| 11 | ("Continuity of Patient Care"/ or "Interprofessional Relations"/) and (drug* or medicat* or pharmaceut* or prescript* or medicines).ti,ab,kf. | 3,924 |
| 12 | "Medication Review"/ or Pharmacy Administration/ or "Drug Utilization Review"/ or Drug Monitoring/ or Medication Adherence/ or Self Medication/ or Medication Reconciliation/ | 58,740 |
| 13 | Pharmacy/ or Community Pharmacy Services/ or Pharmaceutical Services/ or Pharmacies/ or Pharmacists/ or Practice Patterns, Pharmacists'/ | 41,721 |
| 14 | (drug or drugs or medicine* or medication* or pharmaceutic* or "medical history taking").ti,ab,kf. | 2,984,483 |
| 15 | (polypharmacy or polymedication or poly pharmacy or pharmacotherap* or pharmaco therap* or deprescrib* or overprescrib* or pim*).ti,ab,kf. | 75,871 |
| 16 | ((medicat* or pharmac* or prescript* or prescrib* or medicine*) adj2 (manag* or temper* or review* or dosag* or service* or care)).ti,ab,kf. | 70,145 |
| 17 | (pharmacy or pharmacies or pharmacist* or pharmaceutic* service* or pharmaceutic* care).ti,ab,kf. | 88,588 |
| 18 | or/10-17 | 3,285,610 |
| 19 | 8 and 18 | 296,353 |
| 20 | 19 or 9 | 296,718 |
| 21 | "Homes for the Aged"/ or Nursing Homes/ or Assisted Living Facilities/ or Adult Day Care Centers/ or Long-Term Care/ | 69,185 |
| 22 | Independent Living/ or Home Nursing/ or Home Health Nursing/ or Community Health Nursing/ or Health services for the Aged/ | 57,118 |
| 23 | ((aged care or old* people* or old* age* or geriatric) adj (home* or facilit* or residen*)).ti,ab,kf. | 2,998 |
| 24 | (home* for the aged or senior* housing or CCRC* or home* for the elderly or senior residence facilit* or RACF).ti,ab,kf. | 9,789 |
| 25 | (retire* adj (cent* or home* or facilit* or residen* or communit*)).ti,ab,kf. | 1,369 |
| 26 | "residential age* care".ti,ab,kf. | 1,704 |
| 27 | (((nursing or care) adj (home or facilit*)) or ltcf).ti,ab,kf. | 60,063 |
| 28 | ((home or domiciliary) adj (care or healthcare or nurs*)).ti,ab,kf. | 25,425 |
| 29 | or/21-28 | 182,259 |
| 30 | 29 and 20 | 5,679 |
| 31 | limit 30 to yr="2013 -Current" | 3,168 |
| 32 | limit 31 to english language | 3,037 |

**Supplementary Table S2:** Institution websites searched for quality indicators.

| **Country** | **Institution** | **Website URL** | **Relevant QIs identified** |
| --- | --- | --- | --- |
| Australia | Australian Institute of Health and Welfare | https://www.aihw.gov.au/ | Yes |
| Australia | Australian Government Department of Health and Aged Care | https://www.health.gov.au/ | Yes |
| Australia | Australian Government AIHW- Australian institute of Health and Welfare | https://www.gen-agedcaredata.gov.au/topics/quality-in-aged-care | No |
| Australia | RACGP- Royal Australian College of General Practitioners | https://www.racgp.org.au/ | No |
| Australia | NPS- National Prescribing Service MedicineWise | https://www.nps.org.au/ | No |
| Australia | APO- Analysis Policy Observatory-  Australian Commission on Safety and Quality in Health Care Report | https://apo.org.au/ | Yes |
| Australia | VICNISS- Healthcare Associated Infection Surveillance | https://www.vicniss.org.au/ | Yes |
| Australia | NCAS – National Centre for Antimicrobial Stewardship | https://www.ncas-australia.org/ | Yes |
| Australia | Department of Health Victoria | https://www.health.vic.gov.au/residential-aged-care/beyond-compliance | Yes |
| Australia | ERA- Emerging Researchers in Ageing | https://era.edu.au/ | No |
| Canada | CDA- Canada’s Drug Agency | https://www.cadth.ca/ | No |
| Canada | CIHI- Canadian Institute for Health information | https://www.cihi.ca/en | Yes |
| England | NHS England – National Health Service | https://www.england.nhs.uk/ | No |
| Europe | European Directorate for the Quality of Medicines and HealthCare | https://www.edqm.eu/en/ | No |
| Finland | THL Finnish Institute for Health and Welfare - iterRAI | https://thl.fi/en/main-page | Yes |
| Iceland | Icelandic Minimum Data Set | https://island.is/en/interrai-assessment/about-instrument | Yes |
| International | Institute for Healthcare Improvement | https://www.ihi.org/ | No |
| International | ISQua- International Society for Quality in Health Care | https://isqua.org/ | No |
| International | RAND Corporation | https://www.rand.org/ | No |
| International | WHO- World Health Organisation | https://who.int/ | No |
| International | OECD- Organisation for Economic Co-operation and development | https://data-explorer.oecd.org/ | No |
| Ireland | HIQA- Health Information and Quality Authority | https://www.hiqa.ie/ | No |
| Netherland | Zorginstituut Nederland | https://www.zorginzicht.nl/ | No |
| Scottland | Scottish Government National Care Standards | https://www.gov.scot/publications/national-care-standards-guide/ | No |
| Sweden | SveDem- Swedish Dementia Registry | https://www.ucr.uu.se/svedem/in-english | Yes |
| UK | NICE- National Institute for Health and Clinical Excellence | https://www.nice.org.uk/ | Yes |
| UK | UK Government | https://www.gov.uk/ | No |
| USA | CMS – Centers for Medicare and Medicaid Services | https://www.cms.gov/ | Yes |
| USA | CDC NHSN - Centres for Disease Control and Prevention National Healthcare Safety Network | https://www.cdc.gov/nhsn/index.html | Yes |
| USA | AHRQ – Agency for Healthcare Research and Quality | https://qualityindicators.ahrq.gov/resources | No |
| Wales | Welsh Government - National Minimum Standards | https://www.gov.wales/ | No |
