# Supplementary material for: Quality indicators for safe and effective use of medications in long‐term care settings: A systematic review
Source: Br J Clin Pharmacol. 2025 Aug 18;91(11):3054–69. doi: 10.1002/bcp.70242 (PMC12569564; doi:10.1002/bcp.70242)
Supplement: Supplementary file 3 — TABLE S4 Overview of potentially inappropriate medication (PIM) lists (n = 18) that were developed for LTCF or HC, or applied in these populations. [file BCP-91-3054-s003.docx]

**Supplementary Table S4:** Overview of potentially inappropriate medication (PIM) lists (n=18) that were developed for LTCF or HC, or applied in these populations

| **Author, Year** | **PIM List Name** | **Country** | **Population** | **Development/ base of the criteria** | **Validation method** |
| --- | --- | --- | --- | --- | --- |
| **Developed for LTCF & HC** | |  |  |  |  |
| Dumitrescu et al. (2022)^76^ | High-Risk Medication in Home Care Nursing | Europe (Belgium, Germany, Sweden, Netherlands) | People accessing home care | Literature review | Delphi method (3-rounds) |
| Jun et al. (2022)^77^ | PIM-Korea 2022 | Korea | LTCF residents aged ≥65 years | NORGEP-NH, STOPP-START US NH 2017, GheOP3S-Tool V1, Beers criteria 2019, STOPP/START criteria, Turkish criteria (TIME) 2020, PIM Korea 2018, systematic literature review | Modified Delphi method (2-rounds) |
| Nyborg et al. (2015)^78^ | NORGEP-NH  (Norwegian General-Practice-Nursing Home) | Norway | LTCF residents aged ≥ 70 years | NORGEP 2009, literature review | Delphi method (3-rounds) |
| Parsons et al. (2015)^79^ | Parsons’ Criteria | United Kingdom | LTCF residents with advanced dementia | Literature review | Delphi method (3-rounds) |
| Hannou et al. (2014)^80^ | PDE-List  (Preferential list of Drugs adapted to the Elderly in nursing homes) | France | LTCF residents | Beers criteria 2012, PRISCUS 2010, French criteria 2007, literature review, analysis of used medications in LTCFs | Modified Delphi method (2-rounds) |
| **Developed for older adults** | |  |  |  |  |
| American Geriatric Society Beers Criteria Panel (2023)^81^ | AGS Beers criteria | United States | ≥65-year-olds | Beers criteria 2019, systematic literature review | Modified Delphi method (2-rounds) |
| Mann et al. (2023)^82^ | PRISCUS V2.0 | Germany, Austria | ≥65-year-olds | PRISCUS criteria 2010, systematic literature review, | Delphi method (3-rounds) |
| Paulamäki et al. (2023)^83^ | Finnish Meds75+ | Finland | ≥75-year-olds in primary care setting | Beers criteria 2019, STOPP/START V2, EU(7)-PIM, Laroche criteria, review of Finnish prescribing practices and medications | unknown |
| De Schutter et al. (2022) ^84^ | RASP-CARDIO List  (Cardiovascular pharmacotherapy) | Europe (Belgium) | Geriatric patients across healthcare settings requiring cardiovascular medications | RASP list 2014, Beers criteria 2019, STOPP/START V2, European Society of Cardiology Guidelines, literature review | Modified Delphi method (2-rounds) |
| Pazan et al. (2022)^85^ | FORTA List V4  (Fit fOR The Aged) | Germany, Austria, Switzerland | ≥65-year-olds with significant comorbidities^2^ | FORTA V3, literature review | Delphi method (2-rounds) |
| Curtin et al. (2021)^86^ | STOPPFrail V2 | Europe | Frail older people approaching end of life | STOPPFrail V1, systematic literature review | Delphi method (2-rounds) |
| Foubert et al. (2021)^87^ | GheOP^3^S-Tool V2  (Ghent Older People’s Prescriptions Community Pharmacy Screening) | Europe (Belgium) | ≥65-year-olds in primary care | GheOP^3^S V1 1, STOPP/START V2, NORGEP-NH, Beers criteria 2019, literature review | Modified Delphi method (2-rounds) |
| Zhang et al. (2021)^88^ | Hong Kong PIM criteria | Hong Kong | ≥65-year-olds | McLeod criteria 1997, Rancourt criteria 2004, Lindblad criteria 2006, French criteria 2007, Winit-Watjana criteria 2008, NORGEP 2009, PRISCUS criteria 2010, STOPP V2, Beers criteria 2015, literature review | Modified Delphi method (2-rounds) |
| Chang et al. (2019)^89^ | PIM-Taiwan criteria | Taiwan | ≥65-year-olds | PIM-Taiwan criteria 2012, Beers criteria 2015, FORTA 2014, Japan criteria 2016, STOPP V2, systematic literature review | Modified Delphi method (2-rounds) |
| Rodríguez-Pérez (2017)^90^ | LESS-CHRON criteria  (List of Evidence-baSed depreScribing for CHRONic patients) | Spain | Older people with multimorbidity | Systematic literature review, electronic brainstorming | Delphi method (2-rounds) |
| O’Mahony et al. (2015)^91^ | STOPP/START Criteria V2  (Screening Tool of Older Persons’ Prescriptions/ Screening Tool to Alert to Right Treatment) | Europe | ≥65-year-olds | STOPP/START V1 criteria, literature review | Delphi method (2-rounds) |
| Renom-Guiteras et al. (2015)^92^ | EU-7-PIM list | Europe (seven countries) | ≥65-year-olds | PRISCUS criteria 2010, French criteria 2007, STOPP V1, Beers criteria 1997, Beers-Fick 2003, Mcleod criteria 1997, Beers criteria 2012, Micromedex, literature review | Delphi method (2-rounds) |
| Van der Linden et al. (2014)^93^ | RASP List  (Rationalization of home medication by an Adjusted STOPP list in older Patients) | Belgium | Older persons | STOPP V1; literature review | Content Validity Index method (2-rounds) |

Note. *HC* Home care, *LTCF* Long-term care facility, *NH* Nursing home, *V* Version.
